# Triglyceride-glucose index for predicting repeat revascularization and in-stent restenosis in patients with chronic coronary syndrome undergoing percutaneous coronary intervention

**DOI:** 10.1186/s12933-023-01779-7

**Published:** 2023-03-02

**Authors:** Xuantong Guo, Ruihuan Shen, Siyu Yan, Yanni Su, Lihong Ma

**Affiliations:** 1 State Key Laboratory of Cardiovascular Disease, Department of Cardiology, National Clinical Research Center of Cardiovascular Diseases, National Center for Cardiovascular Diseases, Fuwai Hospital, Chinese Academy of Medical Sciences and Peking Union Medical College, Beijing, 100037 China; 2 Beijing Hospital, Department of Cardiology, National Center of Gerontology, Institute of Geriatric Medicine, Chinese Academy of Medical Sciences and Peking Union Medical College, Beijing, 100730 China

**Keywords:** Triglyceride-glucose index, Coronary artery disease, Percutaneous coronary intervention, Repeat revascularization

## Abstract

**Background:**

The triglyceride-glucose (TyG) index, a reliable surrogate indicator of insulin resistance, is independently associated with coronary artery disease of various clinical manifestations. This study aimed to investigate the prognostic value of the TyG index in predicting repeat revascularization and in-stent restenosis (ISR) in chronic coronary syndrome (CCS) patients undergoing percutaneous coronary intervention (PCI).

**Methods:**

A total of 1414 participants were enrolled and divided into groups according to the tertiles of the TyG index. The primary endpoint was a composite of PCI complications, including repeat revascularization and ISR. The associations between the TyG index and the primary endpoint were assessed by multivariable Cox proportional hazards regression analysis with restricted cubic splines (RCS). The TyG index was calculated as Ln (fasting triglycerides (mg/dL) × fasting plasma glucose (mg/dL)/2).

**Results:**

Over a median follow-up of 60 months, 548 (38.76%) patients had experienced at least one primary endpoint event. The follow-up incidence of the primary endpoint increased with the TyG index tertiles. After adjusting for potential confounders, the TyG index was independently associated with the primary endpoint in CCS patients (HR, 1.191; 95% CI 1.038–1.367; P = 0.013). Additionally, the highest tertile of the TyG group was correlated with a 1.319-fold risk of the primary endpoint compared with the lowest tertile of the TyG group (HR, 1.319; 95% CI 1.063–1.637; P = 0.012). Furthermore, a linear and dose–response relationship was observed between the TyG index and the primary endpoint (non-linear P = 0.373, P overall = 0.035).

**Conclusions:**

An increased TyG index was associated with elevated risk for long-term PCI complications, including repeat revascularization and ISR. Our study suggested that the TyG index could be a potent predictor in evaluating the prognosis of CCS patients undergoing PCI.

**Supplementary Information:**

The online version contains supplementary material available at 10.1186/s12933-023-01779-7.

## Background

Coronary artery disease (CAD) has affected 244.11 million individuals worldwide and is the leading cause of death [1]. Chronic coronary syndrome (CCS), previously referred to as stable CAD, contributes to the major population of CAD and encompasses patients with or without history of acute coronary syndrome (ACS) or coronary revascularization [2].

Percutaneous coronary intervention (PCI) with drug-eluting stent (DES) is the most common revascularization strategy utilized in CCS patients. During the past decades, although great advances have been made in the management of CCS patients undergoing PCI-DES, the incidence of repeat revascularization driven by in-stent restenosis (ISR) or progression of non-target lesions persisted over 25% during a 5-year follow-up [3, 4]. Moreover, repeat revascularization was demonstrated to be an independent predictor of myocardial infarction, stroke, and cardiovascular mortality [5]. Of note, despite a reduced risk of ISR observed with DES, ISR remained another major complication associated with adverse cardiac events in patients undergoing DES-PCI [6]. Given that more than 1,000,000 PCIs are performed in China each year, it is of clinical significance to identify risk factors for repeat revascularization and ISR [7].

Insulin resistance (IR) is a clinical state of impaired insulin sensitivity which could result in cardiometabolic alterations, including hyperglycemia, dyslipidemia, and hypertension [8]. Studies discovered that insulin resistance was a strong predictor of CAD and adverse cardiac events in diabetic and nondiabetic subjects [9, 10]. The triglyceride-glucose (TyG) index is a novel surrogate indicator of IR [11]. Growing evidence suggested that the TyG index could independently predict adverse cardiovascular outcomes among CAD cohorts with different clinical manifestations [12–14]. Furthermore, a recent study on the ACS population found that an increased TyG index was positively correlated to ISR [15]. Therefore, the TyG index is now proposed as a potential indicator in identifying patients at high risk of poor prognosis.

In this study, we aimed to address the gap that there is limited evidence regarding the associations of the TyG index for long-term PCI complications, including repeat revascularization and ISR, which might provide clinical benefits for risk stratification and management in CCS patients after DES implantation.

## Methods

### Study population

**Fig. 1:**
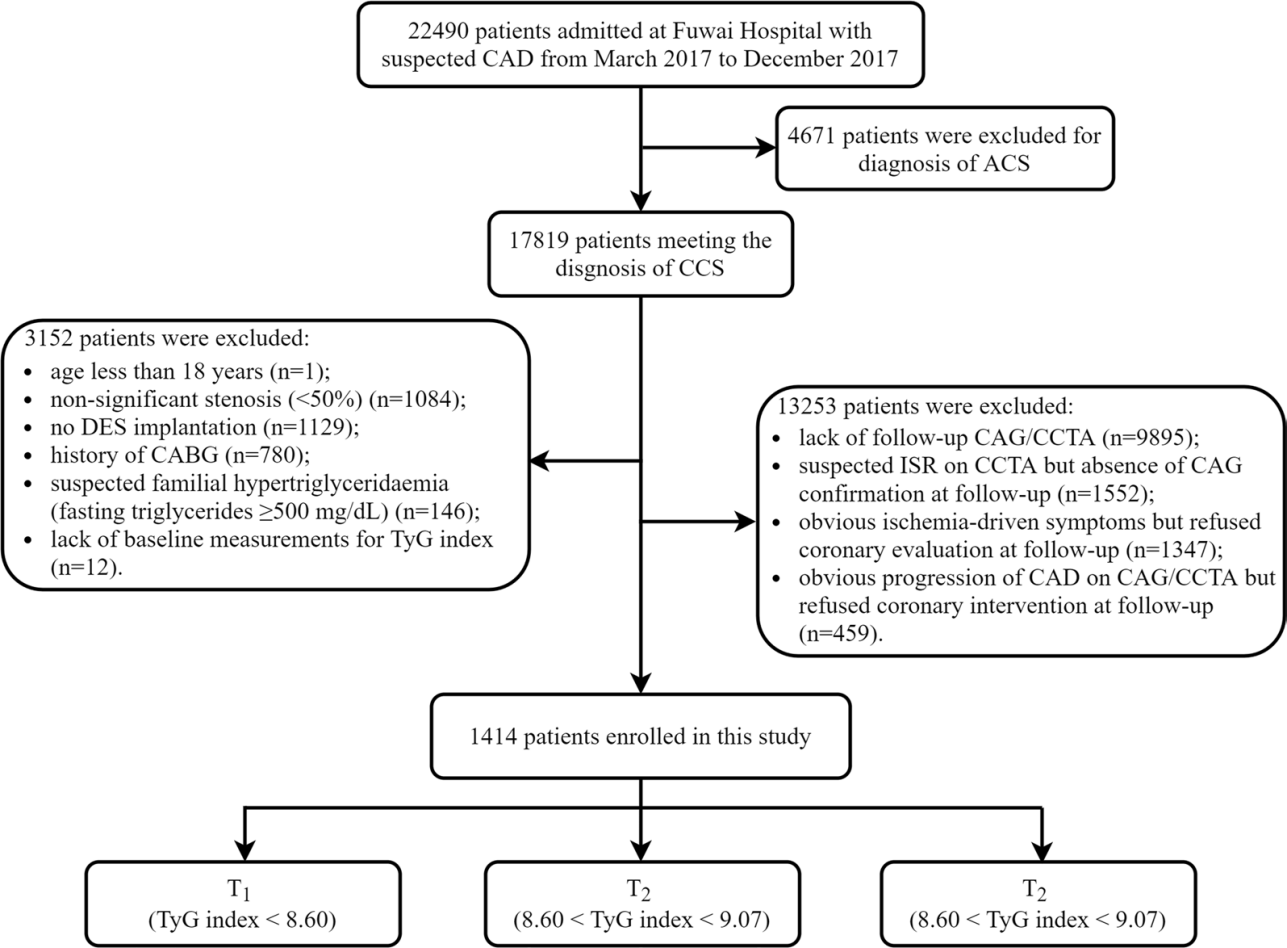
Flowchart of study participants. *ACS* acute coronary syndrome, *CAD* coronary artery disease, *CCS* chronic coronary syndrome, *CABG* coronary artery bypass graft, *CAG* coronary angiography, *CCTA* coronary computed tomography angiography, *DES* drug-eluting stent, *TyG* triglyceride-glucose

This was a retrospective observational study. A total of 1414 consecutive patients diagnosed with CCS in Fuwai Hospital, Chinese National Center for Cardiovascular Diseases, were enrolled from March 2017 to December 2017. The exclusion criteria were as follows: (1) age less than 18 years; (2) non-significant stenosis (< 50%); (3) no DES implantation; (4) history of coronary artery bypass grafting (CABG); (5) lack of follow-up coronary angiography (CAG)/coronary CT angiography (CCTA); (6) lack of baseline measurements for TyG index; (7) suspected familial hypertriglyceridemia (plasma triglycerides ≥ 500 mg/dL); (8) suspected ISR on CCTA but the absence of CAG confirmation at follow-up; (9) obvious ischemia-driven symptoms but refused coronary evaluation at follow-up; (10) obvious progression of CAD on CAG/CCTA but refused coronary intervention at follow-up. The detailed screening process is shown in Fig. 1. This study was performed in line with the Declaration of Helsinki and was authorized by the Ethics Committee of Fuwai Hospital. All participants provided written/oral informed consent.

### Data collection and definitions

Clinical data, including demographics, medical history, and laboratory tests, were collected from medical records. Laboratory tests consisting of fasting blood glucose (FBG), triglyceride (TG), total cholesterol (TC), low-density lipoprotein cholesterol (LDL-C), high-density lipoprotein cholesterol (HDL-C), plasma creatinine (Pcr), high-sensitive C-reactive protein (hs-CRP) and N-terminal pro-B-type natriuretic peptide (NT-proBNP) were performed under standardized instructions and assaying system (LABOSPECT 008, Hitachi, Tokyo, Japan). Glycated hemoglobin (HbA1c) was determined by high-performance liquid chromatography (G8, TOSOH, Tokyo, Japan). All blood samples were collected after overnight fasting before CAG. Therefore, all indicators were at the same temporal window for each participant. The left ventricular ejection fraction (LVEF) was measured by echocardiography using the biplane Simpson method. The angiographic data was obtained from the cardiac catheterization laboratory records.

The definition of CCS complied with the current guideline of the European Society of Cardiology [2]. Body mass index (BMI) was calculated as weight (kg)/height’s square (m^2^). Diabetes mellitus was defined as a past diagnosis with diabetes, FBG ≥ 7.0 mmol/L, HbA1c ≥ 6.5%, or use of antidiabetic drugs. Hypertension was defined as a history of hypertension with anti-hypertensive drugs or repeated blood pressure ≥ 140/90 mmHg. Dyslipidemia was diagnosed as LDL-C ≥ 3.4 mmol/L, HDL-C < 1.0 mmol/L, TG ≥ 1.7 mmol/L, or use of lipid-lowering treatment. As previously reported, the TyG index was calculated as Ln (fasting triglycerides (mg/dL) × fasting plasma glucose (mg/dL)/2) [11]. The estimated glomerular filtration rate (eGFR) was estimated according to the modified Modification of Diet in Renal Disease equations that are based on the Chinese population: 186 × Pcr^−1.154^ × age^−0.203^ × 0.742 (if female) × 1.233 (if Chinese) [16].

### Endpoints and follow-up

The primary endpoint was a composite of PCI complications, including repeat revascularization and ISR. The secondary endpoints were repeat revascularization and ISR. Strategies for repeat revascularization at follow-up comprise DES, bare metal stenting, plain balloon angioplasty, drug-coated balloon angioplasty, and CABG. The repeat revascularization was determined by experienced interventional cardiologists regarding individual risk and patients’ decisions. To avoid counting endpoints in patients with staged PCI, we excluded the records of staged PCI and collected the endpoints under the judgment of experienced cardiologists. The ISR was defined as the presence of significant diameter stenosis (≥ 50%) at the segment inside the stent or involving its 5-mm edges.

All patients underwent follow-up CAG or CCTA in Fuwai Hospital after the baseline successful PCI. The follow-up period lasted until October 2022. Considering the high specificity of CCTA, we mainly included patients diagnosed with the absence of ISR by CCTA. For the patients with suspected ISR, a CAG confirmation was required. Importantly, the follow-up CAG and CCTA were interpreted by a panel of dependent experienced radiologists and cardiologists. The coronary revascularization and periprocedural management were performed according to current guidelines and regulations of our center. All patients have received standard secondary prevention recommended by current guidelines [2].

### Statistical analysis

Continuous variables were described as mean ± standard deviation if consistent with a normal distribution, otherwise as median and interquartile range (IQR). Categorical variables were presented as numbers and percentages (%). All participants were stratified into three groups: T_1_ (TyG index < 8.60), T_2_ (8.60 ≤ TyG index < 9.07), and T_3_ (TyG index ≥ 9.07) in accordance with the TyG index tertiles. The Analysis of variance was applied to analyze the difference in continuous variables between groups, while the Kruskal–Wallis test was used to analyze the difference in categorical variables.

Multiple imputation was used in the imputation of the missing data by Gibbs sampling [17, 18]. The target variables containing missing data were predicted based on the whole dataset. The process was iterated until all the missing values converged, and five imputed datasets with chained equations were created using R multivariate imputation by chained equation software package [19].

Log-rank tests and Kaplan–Meier methods were performed to explore differences in event rates between TyG tertiles and plot time-to-event curves. P values for pairwise comparisons were corrected for multiple testing by the Benjamin-Hochberg algorithm. The Cox proportional hazards regression analysis was used to estimate the hazard ratio (HR) and the 95% confidence interval (CI) of the TyG index in developing the primary and secondary endpoints. In the multivariable model, the following confounders were chosen considering the clinical importance: age (continuous), sex, BMI, previous PCI, presence of peripheral artery disease (PAD), presence of multivessel CAD, eGFR (continuous), hs-CRP (continuous), presence of lesion’s length ≥ 20 mm, stent length (continuous). Trend analyses were conducted by entering the tertiles of the TyG index as a continuous variable and rerunning the corresponding regression models. Moreover, the RCS were used to examine the possible nonlinear relationships of the TyG index with the primary and secondary endpoints. To balance best fit and overfitting in the RCS, the Akaike information criterion was used [20]. The potential non-linearity of the RCS was tested through a likelihood ratio test that compares the model presenting only a linear term with the model presenting linear and cubic spline terms [18, 21, 22]. The median of the TyG index was assigned as the reference value.

All statistical analyses were performed in R software (version 4.1.2). A two‐sided P value < 0.05 was considered statistically significant.

## Results

### Baseline characteristics

The average age of the 1414 CCS participants undergoing DES-PCI was 58.04 ± 0.25 years, and 1103 (78.00%) were men. Compared with the T_1_ group, T_2_ and T_3_ group participants tended to have higher BMI (P < 0.001). The proportions of cardiometabolic risk factors, including diabetes, hypertension, family history of CAD, and the use of insulin and oral hypoglycemic drugs, were higher in the T_3_ group than in the other two groups (all P < 0.05). Moreover, the participants of the T_3_ group had higher TC, LDL-C, triglycerides, FPG, HbA1c, and Pcr (all P < 0.001) (Table 1).

**Table 1:** Baseline characteristics according to tertiles of the TyG index

Variable	T_1_ (n = 467)	T_2_ (n = 466)	T_3_ (n = 481)	P-value
Demographics				
Age (years)	58.94 ± 9.55	58.34 ± 9.80	56.86 ± 8.52	0.002
Male sex, n (%)	371 (79.44)	365 (78.33)	367 (76.30)	0.495
BMI (kg/m^2^)	24.80 (23.04, 26.73)	26.12 (24.22, 28.37)	26.08 (24.54, 28.01)	< 0.001
Risk factors				
Cigarette smoking, n (%)	281 (60.17)	280 (60.09)	297 (61.75)	0.840
Diabetes, n (%)	125 (26.77)	169 (36.27)	303 (62.99)	< 0.001
Hypertension, n (%)	272 (58.24)	301 (64.59)	333 (69.23)	0.002
Dyslipidemia, n (%)	464 (99.36)	465 (99.79)	481 (100.00)	0.167
Previous MI, n (%)	66 (14.13)	69 (14.81)	72 (14.97)	0.929
Previous stroke, n (%)	46 (9.85)	52 (11.16)	63 (13.10)	0.285
Previous PCI, n (%)	133 (28.48)	132 (28.33)	131 (27.23)	0.897
PAD, n (%)	75 (16.06)	62 (13.30)	57 (11.85)	0.161
Family history of CAD, n (%)	80 (17.13)	72 (15.45)	108 (22.45)	0.015
Clinical presentations				
Multi-vessel CAD, n (%)	364 (77.94)	380 (81.55)	396 (82.33)	0.1926
LVEF (%)	64 (61, 66)	64 (60, 66)	64 (60, 66)	0.852
Laboratory measurements				
TC (mmol/L)	3.65 ± 0.84	4.06 ± 0.96	4.45 ± 1.14	< 0.001
LDL-C (mmol/L)	2.16 ± 0.70	2.50 ± 0.85	2.58 ± 0.92	< 0.001
HDL-C (mmol/L)	1.18 ± 0.30	1.10 ± 0.30	1.01 ± 0.28	< 0.001
Triglycerides (mmol/L)	0.98 ± 0.22	1.51 ± 0.32	2.74 ± 1.60	< 0.001
FPG (mmol/L)	5.28 ± 0.88	5.94 ± 1.39	7.57 ± 2.83	< 0.001
HbA1c (%)	5.92 ± 0.78	6.24 ± 0.98	6.95 ± 1.47	< 0.001
Pcr (μmol/L)	79.38 ± 14.46	81.71 ± 16.40	83.59 ± 21.41	0.001
eGFR (mL/min per 1.73 m^2^)	112.44 (100.22, 127.78)	110.84 (98.58, 124.76)	108.33 (93.36, 125.47)	0.005
NT-proBNP (pg/mL)	194.31 ± 428.14	163.92 ± 296.40	140.80 ± 256.45	0.048
hs-CRP (mg/L)	3.58 ± 6.36	4.30 ± 6.57	4.27 ± 4.74	0.112
Medications at discharge				
DAPT, n (%)	464 (99.36)	464 (99.57)	476 (98.96)	0.523
Statins, n (%)	454 (97.22)	458 (98.28)	474 (98.54)	0.301
Dual-lipid lowering therapy, n (%)	15 (3.21)	19 (4.08)	28 (5.82)	0.135
ACEI/ARBs, n (%)	199 (42.61)	244 (52.36)	264 (54.89)	< 0.001
β-Blockers	372 (79.66)	393 (84.33)	418 (86.90)	0.009
Insulin, n (%)	36 (7.71)	32 (6.87)	87 (18.09)	< 0.001
Oral hypoglycemic drugs, n (%)	74 (15.85)	100 (21.46)	193 (40.12)	< 0.001
Angiographic findings				
Restenotic lesions, n (%)	20 (4.28)	26 (5.58)	27 (5.61)	0.576
Chronic total occlusions, n (%)	53 (11.35)	74 (15.88)	64 (13.31)	0.1272
Lesions > 20 mm long, n (%)	321 (68.74)	335 (71.89)	344 (71.52)	0.511
Number of stents	2 (1, 2)	2 (1, 2.75)	2 (1, 2)	0.076
Length of stent (mm)	30.00 (20.00, 45.50)	33.00 (21.00, 51.00)	30.00 (22.00, 46.00)	0.231

*ACEI* angiotensin-converting enzyme inhibitor, *ARBs* angiotensin receptor blockers, *BMI* body mass index, *CAD* coronary artery disease, *DAPT* dual antiplatelet therapy, *eGFR* estimated glomerular filtration rate, *FPG* fasting plasma glucose, *HDL-C* high-density lipoprotein-cholesterol, *HbA1c* glycated hemoglobin A1c, *hs-CRP* hypersensitive C-reactive protein, *LVEF* left ventricular ejection fraction, *LDL-C* low-density lipoprotein-cholesterol, *MI* myocardial infarction, *NT-proBNP* N-terminal pro-B-type natriuretic peptide, *PCI* percutaneous coronary intervention, *PAD* peripheral arterial disease, *Pcr* plasma creatine, *TC* total cholesterol, *TyG* triglyceride-glucose

Table 2 presents the baseline characteristics according to the primary endpoint. A total of 548 participants were observed experiencing repeat revascularization and/or ISR during the follow-up. Compared with participants without endpoints, participants with an endpoint event had higher BMI and greater proportions of diabetes, previous MI, previous PCI, multi-vessel CAD, and use of insulin and oral hypoglycemic drugs (all P < 0.05). Besides. participants with an endpoint event showed elevated concentrations of LDL-C, FPG, HbA1c, and statistically significant different LVEF (all P < 0.05). Furthermore, participants experiencing a primary endpoint had higher levels of TyG, and the participants with an endpoint event tended to be in the T_3_ group (both P < 0.05). Compared to those without an endpoint event, patients with the primary endpoint had higher proportions of restenotic lesions, chronic total occlusions, lesions > 20 mm long, number of stents, and smaller diameter of stent (all P < 0.05).

**Table 2:** Baseline characteristics according to the primary endpoint

Variable	No such events (n = 866)	Primary endpoint (n = 548)	P-value
Demographics			
Age (years)	58.21 ± 9.35	57.76 ± 9.31	0.374
Male sex, n (%)	668 (77.14)	435 (79.38)	0.354
BMI (kg/m^2^)	25.64 (23.80, 27.64)	25.95 (24.22, 28.29)	0.004
Risk factors			
Cigarette smoking, n (%)	521 (60.16)	337 (61.50)	0.657
Diabetes, n (%)	329 (37.99)	268 (48.91)	< 0.001
Hypertension, n (%)	550 (63.51)	356 (64.96)	0.619
Dyslipidemia, n (%)	864 (99.77)	546 (99.64)	1
Previous MI, n (%)	104 (12.01)	103 (18.80)	0.001
Previous stroke, n (%)	96 (11.09)	65 (11.86)	0.718
Previous PCI, n (%)	198 (22.86)	198 (36.13)	< 0.001
PAD, n (%)	120 (13.86)	74 (13.50)	0.913
Family history of CAD, n (%)	168 (19.40)	92 (16.79)	0.244
Clinical presentations			
Multi-vessel CAD, n (%)	652 (75.29)	488 (89.05)	< 0.001
LVEF (%)	64 (61, 66)	64 (60, 66)	0.010
Laboratory measurements			
TC (mmol/L)	4.02 ± 1.03	4.10 ± 1.06	0.159
LDL-C (mmol/L)	2.38 ± 0.83	2.47 ± 0.88	0.049
HDL-C (mmol/L)	1.11 ± 0.30	1.07 ± 0.29	0.003
Triglycerides (mmol/L)	1.71 ± 1.22	1.82 ± 1.20	0.129
FPG (mmol/L)	6.09 ± 1.93	6.57 ± 2.39	< 0.001
HbA1c (%)	6.24 ± 1.06	6.60 ± 1.36	< 0.001
Pcr (μmol/L)	81.24 ± 15.59	82.11 ± 20.79	0.370
eGFR (mL/min per 1.73 m^2^)	110.43 (97.32, 123.70)	111.88 (95.43, 128.43)	0.309
NT-proBNP (pg/mL)	156.03 ± 329.01	181.99 ± 343.80	0.156
hs-CRP (mg/L)	3.83 ± 5.01	4.40 ± 7.15	0.079
Medications at discharge			
DAPT, n (%)	859 (99.19)	545 (99.45)	0.807
Statins, n (%)	849 (98.04)	537 (97.99)	1.000
Dual-lipid lowering therapy, n (%)	36 (4.16)	26 (4.74)	0.695
ACEI/ARBs, n (%)	414 (47.81)	293 (53.47)	0.043
β-Blockers	697 (80.48)	486 (88.69)	0.0001
Insulin, n (%)	69 (7.97)	86 (15.69)	< 0.001
Oral hypoglycemic drugs, n (%)	202 (23.33)	165 (30.11)	0.006
Angiographic findings			
Restenotic lesions, n (%)	29 (3.35)	44 (8.03)	< 0.001
Chronic total occlusions, n (%)	94 (10.85)	97 (17.70)	< 0.001
Lesions > 20 mm long, n (%)	588 (67.90)	412 (75.18)	0.004
Number of stents	2 (1, 2)	2 (1, 3)	< 0.001
Length of stent (mm)	26.50 (16.00, 40.00)	30.00 (20.00, 50.00)	< 0.001
TyG index	8.80 (8.45, 9.20)	8.90 (8.55, 9.34)	< 0.001
TyG tertiles			0.001
T_1_, n (%)	316 (67.67)	151 (32.33)	
T_2_, n (%)	279 (59.87)	187 (40.13)	
T_3_, n (%)	271 (56.34)	210 (43.66)	

*ACEI* angiotensin-converting enzyme inhibitor, *ARBs* angiotensin receptor blockers, *BMI* body mass index, *CAD* coronary artery disease, *DAPT* dual antiplatelet therapy, *eGFR* estimated glomerular filtration rate, *FPG* fasting plasma glucose, *HDL-C* high-density lipoprotein-cholesterol, *HbA1c* glycated hemoglobin A1c, *hs-CRP* hypersensitive C-reactive protein, *LVEF* left ventricular ejection fraction, *LDL-C* low-density lipoprotein-cholesterol, *MI* myocardial infarction, *NT-proBNP* N-terminal pro-B-type natriuretic peptide, *PCI* percutaneous coronary intervention, *PAD* peripheral arterial disease, *Pcr* plasma creatine, *TC* total cholesterol, *TyG* triglyceride-glucose

The respective baseline information according to the secondary endpoints is shown in Additional file 1: Tables S1–S2. More details about the angiographic findings and medications at charge among the TyG index tertiles are shown in Additional file 1: Table S3.

### Association between the TyG index and the primary endpoint

**Fig. 2:**
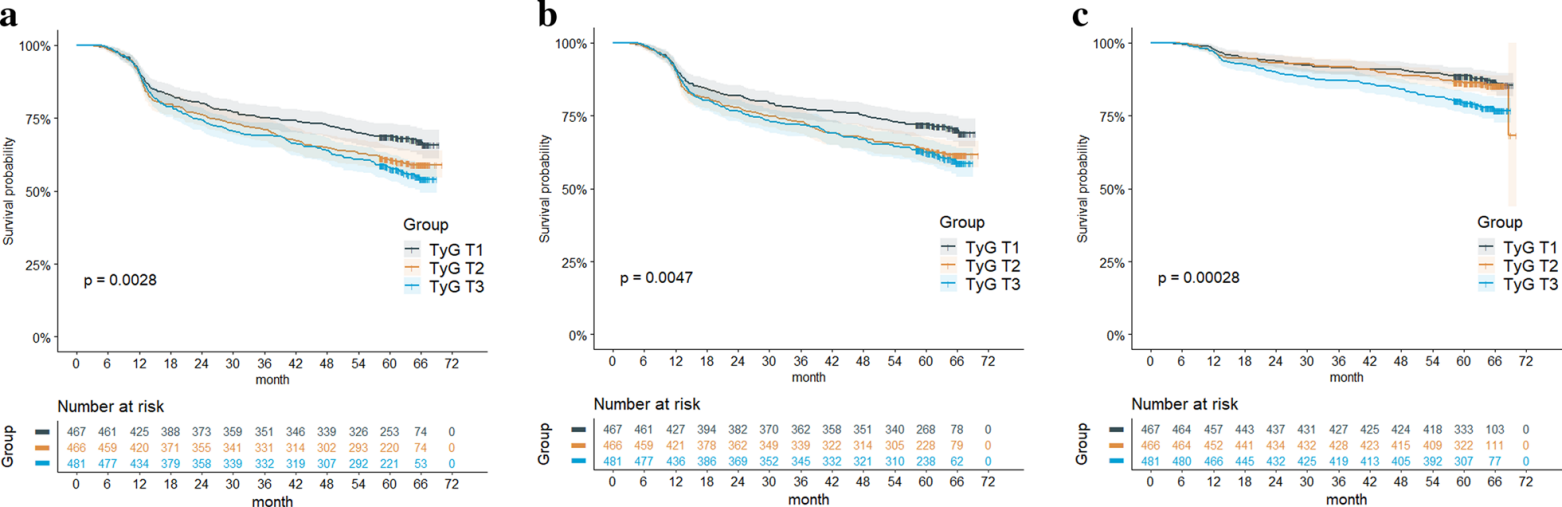
Kaplan–Meier analyses for the incidences of the primary endpoint and secondary endpoints among the TyG index tertiles. **a** The primary endpoint including repeat revascularization and/or ISR; **b** repeat revascularization; **c** ISR. *ISR* in-stent restenosis, *TyG* triglyceride-glucose

The median follow-up duration of the patients for the primary endpoint was 60 months (IQR 56–64 months). During the follow-up period, 548 (38.76%) patients had the primary endpoint. Results of the Kaplan–Meier survival analyses are presented in Fig. 2. It’s indicated that the incidence of the primary endpoint in T_3_ was significantly higher than that in the T_1_ (log-rank test, overall P = 0.0028, adjusted pairwise P values between T_1_ and T_3_ = 0.0021), which was driven by the increase in repeat revascularization (log-rank test, overall P = 0.0047, adjusted pairwise P values between T_1_ and T_3_ = 0.0058) as well as ISR (log-rank test, overall P < 0.001, adjusted pairwise P values between T_1_ and T_3_ = 0.0007). Table 3 describes the results of the multivariable Cox proportional hazards regression analysis. The unadjusted model 1 indicated that the TyG index was statistically significantly associated with the primary endpoint, and the T_3_ was at elevated risk for an endpoint event. After adjusting for age, sex, BMI, previous PCI, presence of PAD, presence of multivessel CAD, hs-CRP, eGFR, and presence of lesion’s length ≥ 20 mm in model 2, the TyG index as a continuous variable was an independent predictor for the primary endpoint (HR, 1.201; 95% CI 1.047–1.377; P = 0.009). Taking the T_1_ as a reference, the risk of the primary endpoint was 1.338-fold higher (HR, 1.338; 95% CI 1.078–1.659; P = 0.008) in the T_3_. After further adjusting for stent length in model 3, the TyG index as a continuous variable remained independently associated with the primary endpoint (HR, 1.191; 95% CI 1.038–1.367; P = 0.013). Using T_1_ as the reference, the risk of participants experiencing the primary endpoint increased by 31.9% (HR, 1.319; 95% CI 1.063–1.637; P = 0.012) in the T_3_. The trend analyses for the three models were all statistically significant (all P for trend < 0.05).

**Table 3:** Associations between the TyG index and the primary endpoint

	Model 1			Model 2			Model 3		
	HR	95% CI	P-value	HR	95%CI	P-value	HR	95% CI	P-value
TyG index	1.274	1.114–1.456	< 0.001	1.201	1.047–1.377	0.009	1.191	1.038–1.367	0.013
TyG tertiles									
T_1_	Reference			Reference			Reference		
T_2_	1.301	1.050–1.612	0.016	1.212	0.974–1.509	0.085	1.194	0.959–1.487	0.112
T_3_	1.432	1.162–1.765	< 0.001	1.338	1.078–1.659	0.008	1.319	1.063–1.637	0.012
P for trend			< 0.001			0.009			0.013

Model 1: unadjusted

Model 2: adjusted for age, sex, BMI, previous PCI, presence of PAD, presence of multivessel CAD, high-sensitivity CRP, eGFR, presence of lesion’s length ≥ 20 mm

Model 3: adjusted for age, sex, BMI, previous PCI, presence of PAD, presence of multivessel CAD, high-sensitivity CRP, eGFR, presence of lesion’s length ≥ 20 mm, stent length

*BMI* body mass index, *CI* confidence interval, *CAD* coronary artery disease, *CRP* C-reactive protein, *eGFR* estimated glomerular filtration rate, *HR* hazard ratio, *PCI* percutaneous coronary intervention, *PAD* peripheral artery disease, *TyG* triglyceride-glucose

**Fig. 3:**
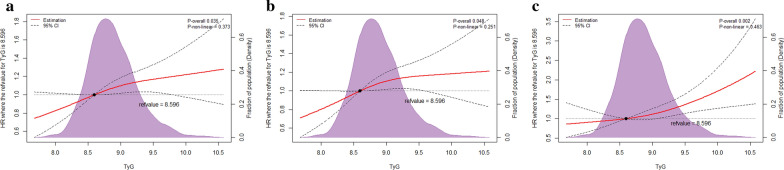
Restricted cubic splines for the adjusted dose–response association of the TyG index for the primary and secondary endpoints. Data of the TyG index for repeat revascularization was fitted with a linear regression model using restricted cubic spines with three knots at the 5th, 50th, and 95th percentiles of the TyG index. Y-axis represents the odds ratio, and the dashed lines are 95% confidence intervals. **a** The primary endpoint including repeat revascularization and/or ISR; **b** repeat revascularization; **c** ISR. *CI* confidence interval, *ISR* in-stent restenosis, *OR* odds ratio, *TyG* triglyceride-glucose

The association of the TyG index with the primary endpoint in model 3 was visualized in Fig. 3a using RCS. A linear and dose–response relationship between the TyG index and the primary endpoint was observed (non-linear P = 0.373, P overall = 0.035).

### Association between the TyG index and the secondary endpoint

A sum of 499 participants experienced repeat revascularization. The TyG index, as a continuous variable, was found to be significantly associated with repeat revascularization in model 1 and independently predicted repeat revascularization in model 2 and model 3 (all P < 0.05). Using the T_1_ as the reference, each additional unit increase in the TyG index was found to increase the risk for repeat revascularization by 29.2% (HR, 1.292; 95% CI 1.030–1.622; P = 0.027) and 27.4% (HR, 1.274; 95% CI 1.015–1.600; P = 0.037) in model 2 and model 3, respectively. Besides, there was statistical significance in the trend analyses (all P for trend < 0.05). The relation of the TyG index with repeat revascularization in model 3 was fitted by RCS (Fig. 3b). Similarly, the TyG index was related to the risk of repeat revascularization in a linear and dose–response relationship (non-linear P = 0.251, P overall = 0.048).

There were 230 participants having ISR. The overall association between the TyG index and the risk of ISR was statistically significant (P overall = 0.002). After adjusting for potential confounders in model 3, the result of the RCS revealed a linear association between the TyG index and ISR (non-linear P = 0.463) (Fig. 3c). Moreover, the TyG index at 8.596 was indicated to be the reference value for the dose–response relationship that participants with a TyG index higher than 8.596 were associated with higher risk for ISR (HR, 1.738; 95% CI 1.250–2.417; P = 0.001). Detailed results of Cox regression analyses for the secondary endpoints are shown in Additional file 1: Tables S4, S5.

## Discussion

This is the first study to investigate the prognostic value of the TyG index in the CCS population. In the present study, we observed a significant correlation between the TyG index and the incidence of the primary endpoint, including repeat revascularization and ISR, in the CCS patients undergoing DES-PCI. After adjusting for potential confounding risk factors, an independent association remained. Additionally, the highest tertile of the TyG group was correlated with a 1.319-fold risk of the primary endpoint compared with the lowest tertile of the TyG group. Moreover, a linear and dose–response relationship was detected between the TyG index and the risk of the primary endpoint. These findings suggested that the TyG index could be a promising predictor in evaluating the prognosis of CCS patients undergoing DES-PCI, thus benefiting risk stratification and management.

Although repeat revascularization was a controversial outcome in clinical trials for its subjective and biased nature, previous studies demonstrated that it was significantly associated with elevated risk for mortality and morbidity in the short term and composite safety events in the long term [23]. Moreover, reductions in repeat revascularization were shown to translate into improved prognosis [4]. Therefore, it’s of great significance in discussing repeat revascularization, which might provide insights into risk modification. The TyG index was found positively associated with ischemia-driven revascularization and target vessel revascularization in ACS patients [24, 25]. Based on this, this study was designed to focus on the overall repeat revascularization in the CCS population, which reflects not only the target lesion failure but also the progression of non-target lesions. We have confirmed a significant association between the TyG index and repeat revascularization. Additionally, the TyG index was correlated with the risk of repeat revascularization in a linear and dose–response way.

ISR is a delayed complication of stenting [26]. Observational studies discovered that patients with ISR were more prone to develop ACS and adverse cardiac events at follow-up [27]. Recently, the TyG index was identified as an independent predictor of ISR in ACS patients, indicating a prospect for the TyG index in ISR evaluation [15]. Consistently, our study discovered that the TyG index was significantly associated with ISR in a linear relationship after adjusting for all potential cofounders, in which a TyG index of 8.596 may serve as a cut-off value.

Putative mechanisms underlying the associations of the TyG index with repeat revascularization and ISR are not fully elucidated, although several underlying mechanisms have been proposed. Firstly, given that diagnostic studies suggested an outstanding accuracy of the TyG index in predicting IR, it’s speculated that the TyG index is a reliable surrogate indicator of IR which could reflect the pro-atherogenic roles of IR [28]. Previous experimental data established that IR could promote atherosclerosis and neointimal hyperplasia by activating the mitogen-activated protein kinase signaling pathway, resulting in vascular smooth muscle cell proliferation, inflammatory aggravation, and lipogenesis stimulation [29, 30]. Besides, IR could induce plaque development by affecting nitric oxide production (endothelial function) [31]. Observational studies verified that IR was independently associated with CAD incidence and ISR among patients with or without diabetes [10, 32]. Secondly, the chronic inflammation, disturbed lipid, and glucose homeostasis following IR may participate in the pathophysiology of repeat revascularization and ISR [33]. Still, the roles of the TyG index in repeat revascularization and ISR warrant further research.

Currently, the prognostic value of the TyG index is widely investigated in CAD. In a meta-analysis of eight cohorts involving 5,731,294 participants, the TyG index was independently associated with an increased incidence of CAD [34]. Another population-based study with a sample size of 5,593,134 demonstrated an elevated risk for MI in participants with the highest TyG index quartile [35]. Clinical investigations further supported that the TyG index was a reliable predictor of adverse coronary events and all-cause death in ACS cohorts [13]. Moreover, the TyG index was explored in various vascular diseases such as subclinical atherosclerosis, hypertension, coronary calcification, and arterial stiffness [36–38]. In this context, the present study powered by repeat revascularization and ISR among the CCS population has extended the association between the TyG index and CAD, indicating that the TyG index could serve as a potent prognostic indicator for risk stratification in CCS patients undergoing DES-PCI.

Our study had several limitations. First, the study excluded CCS patients with CABG, which might affect the generalizability of the findings. However, given that different incentives for repeat revascularization have been reported in patients with PCI or CABG, the population in our study was selected to avoid bias and emphasize the prognosis of DES-PCI. Second, there might be collection bias as we excluded patients without follow-up CAG or CCTA. Finally, the TyG index in our study was measured only once at the baseline hence the change in the TyG index during follow-up could not be taken into consideration. Therefore, a longitudinal study that monitors long-term levels of the TyG index after discharge might need to validate the finding.

## Conclusion

Our study indicated that an increased TyG index was independently and significantly associated with a higher risk of long-term PCI complications, including repeat revascularization and ISR among CCS patients undergoing DES-PCI. Additionally, a linear and dose–response relationship was observed between the TyG index and the risk of developing repeat revascularization and ISR. Therefore, our study suggested that the assessment of the TyG index might help identify patients with high risk in the early stages, thus benefiting CCS management. Further prospective cohort studies are needed to confirm our findings.

## Supplementary Information

**Additional file 1.**

## Abbreviations

ACS: Acute coronary syndrome
BMI: Body mass index
CAD: Coronary artery disease
CCS: Chronic coronary syndrome
CABG: Coronary artery bypass grafting
CAG: Coronary angiography
CCTA: Coronary CT angiography
Cis: Confidence intervals
DES: Drug-eluting stent
eGFR: Estimated glomerular filtration rate
FBG: Fasting blood glucose
HDL-C: High-density lipoprotein cholesterol
hs-CRP: High-sensitive C-reactive protein
HbA1c: Glycated hemoglobin
HR: Hazard ratio
ISR: In-stent restenosis
IQR: Interquartile range
IR: Insulin resistance
LDL-C: Low-density lipoprotein cholesterol
LVEF: Left ventricular ejection fraction
NT-proBNP: N-terminal pro-B-type natriuretic peptide
OR: Odd ratio
PCI: Percutaneous coronary intervention
Pcr: Plasma creatine
PAD: Peripheral artery disease
RCS: Restricted cubic splines
TG: Triglyceride
TC: Total cholesterol

## Declarations

### Availability of data and materials

The datasets used and analyzed during the current study are available from the corresponding author upon reasonable request.
