# Supplementary material for: Triglyceride-glucose index for predicting repeat revascularization and in-stent restenosis in patients with chronic coronary syndrome undergoing percutaneous coronary intervention
Source: Cardiovasc Diabetol. 2023 Mar 2;22:43. doi: 10.1186/s12933-023-01779-7 (PMC9983161; doi:10.1186/s12933-023-01779-7)
Supplement: Supplementary file 1 — Additional file 1. [file 12933_2023_1779_MOESM1_ESM.doc]

**ADDITIONAL FILE 1**

Baseline information according to repeat revascularization and in-stent restenosis is shown in **Tables S1–S2**. Details of the angiographic findings and medication at discharge according to the TyG index tertiles are presented in **Table S3.** Results of the multivariable Cox hazard analysis for the secondary endpoints are shown in **Tables S4–S5.**

Continuous variables were described as mean ± standard deviation if consistent with a normal distribution, otherwise as median and interquartile range. Categorical variables were presented as numbers and percentages (%). The Analysis of variance was applied to analyze the difference in continuous variables between groups, while the Kruskal–Wallis test was used to analyze the difference in categorical variables. Multiple imputation was used in the imputation of the missing data by Gibbs sampling. Log-rank tests and Kaplan–Meier methods were performed to explore differences in event rates between TyG tertiles and plot time-to-event curves. P values for pairwise comparisons were corrected for multiple testing by the Benjamin-Hochberg algorithm. The Cox proportional hazards regression analysis was used to estimate the hazard ratio (HR) and the 95% confidence interval (CI). Trend analyses were conducted by entering the tertiles of the TyG index as a continuous variable and rerunning the corresponding regression models. All statistical analyses were performed in R software (version 4.1.2). A two‐sided P value < 0.05 was considered statistically significant.

**Table S1**. Baseline characteristics according to repeat revascularization

| Variable | No such events  (n=915) | Repeat revascularization  (n=499) | P-value |
| --- | --- | --- | --- |
| Demographics |  |  |  |
| Age (years) | 58.15±9.29 | 57.82±9.42 | 0.526 |
| Male sex, n (%) | 705 (77.05) | 398 (79.76) | 0.268 |
| BMI (kg/m^2^) | 25.64 (23.80, 27.68) | 26.03 (24.22, 28.32) | 0.002 |
| Risk factors |  |  |  |
| Cigarette smoking, n (%) | 550 (60.11) | 308 (61.72) | 0.591 |
| Diabetes, n (%) | 355 (38.80) | 242 (48.50) | <0.001 |
| Hypertension, n (%) | 579 (63.28) | 327 (65.53) | 0.432 |
| Dyslipidemia, n (%) | 913 (99.78) | 497 (99.60) | 0.926 |
| Previous MI, n (%) | 119 (13.01) | 88 (17.64) | 0.023 |
| Previous stroke, n (%) | 101 (11.04) | 60 (12.02) | 0.638 |
| Previous PCI, n (%) | 228 (24.92) | 168 (33.67) | <0.001 |
| PAD, n (%) | 129 (14.10) | 65 (13.03) | 0.632 |
| Family history of CAD, n (%) | 176 (19.23) | 84 (16.83) | 0.297 |
| Clinical presentations |  |  |  |
| Multi-vessel CAD, n (%) | 692 (75.63) | 448 (89.78) | <0.001 |
| LVEF (%) | 64 (61, 66) | 64 (60, 66) | 0.007 |
| Laboratory measurements |  |  |  |
| TC (mmol/L) | 4.02±1.02 | 4.12±1.08 | 0.092 |
| LDL-C (mmol/L) | 2.37±0.83 | 2.49±0.89 | 0.011 |
| HDL-C (mmol/L) | 1.11±0.30 | 1.06±0.29 | 0.001 |
| Triglycerides (mmol/L) | 1.72±1.22 | 1.81±1.19 | 0.203 |
| FPG (mmol/L) | 6.11±1.96 | 6.58±2.39 | <0.001 |
| HbA1c (%) | 6.25±1.10 | 6.60±1.34 | <0.001 |
| Pcr, (μmol/L) | 81.03±15.46 | 82.58±21.38 | 0.119 |
| eGFR, (ml/min per 1.73m^2^) | 110.78 (97.60, 124.11) | 111.55 (94.97, 128.10) | 0.672 |
| NT-proBNP, (pg/ml) | 154.48±321.92 | 187.39±356.95 | 0.078 |
| hs-CRP, (mg/L) | 3.82±5.01 | 4.48±7.33 | 0.045 |
| Medications at discharge |  |  |  |
| DAPT, n (%) | 912 (99.67) | 499 (100.00) | 0.499 |
| Statins, n (%) | 898 (98.14) | 488 (97.80) | 0.805 |
| Dual-lipid lowering therapy, n (%) | 40 (4.37) | 22 (4.41) | 1.000 |
| ACEI/ARBs, n (%) | 438 (47.87) | 269 (53.91) | 0.035 |
| β-blockers | 739 (80.77) | 444 (88.98) | <0.001 |
| Insulin, n (%) | 75 (8.20) | 80 (16.03) | <0.001 |
| Oral hypoglycemic drugs, n (%) | 215 (23.50) | 152 (30.46) | 0.005 |
| Angiographic findings |  |  |  |
| Restenotic lesions, n (%) | 42 (4.59) | 31 (6.21) | 0.233 |
| Chronic total occlusions, n (%) | 104 (11.37) | 87 (17.43) | 0.002 |
| Lesions>20 mm long, n (%) | 621 (67.87) | 379 (75.95) | 0.002 |
| Number of stents | 2 (1, 2) | 2 (1, 3) | <0.001 |
| Length of stent, (mm) | 30.00 (20.00, 45.00) | 33.00 (23.00, 54.00) | <0.001 |
| TyG index | 8.81 (8.45, 9.21) | 8.90 (8.56, 9.34) | <0.001 |
| TyG tertiles |  |  | 0.003 |
| T_1_, n (%) | 331 (70.88) | 136 (29.12) |  |
| T_2_, n (%) | 291 (62.45) | 175 (37.55) |  |
| T_3_, n (%) | 293 (60.91) | 188 (39.09) |  |

ACEI, angiotensin-converting enzyme inhibitor; ARBs, angiotensin receptor blockers; BMI, body mass index; CAD, coronary artery disease; DAPT, dual antiplatelet therapy; eGFR, estimated glomerular filtration rate; FPG, fasting plasma glucose; HDL-C, high-density lipoprotein-cholesterol; HbA1c, glycated hemoglobin A1c; hs-CRP, hypersensitive C-reactive protein; LVEF, left ventricular ejection fraction; LDL-C, low-density lipoprotein-cholesterol; MI, myocardial infarction; NT-proBNP, N-terminal pro-B-type natriuretic peptide; PCI, percutaneous coronary intervention; PAD, peripheral arterial disease; Pcr, plasma creatine; TC, total cholesterol; TyG, triglyceride–glucose.

**Table S2**. Baseline characteristics according to in-stent restenosis

| Variable | No such events  (n=1184) | In-stent restenosis  (n=230) | P-value |
| --- | --- | --- | --- |
| Demographics |  |  |  |
| Age (years) | 58.19±9.41 | 57.23±8.93 | 0.150 |
| Male sex, n (%) | 922 (77.87) | 181 (78.70) | 0.850 |
| BMI (kg/m^2^) | 25.71 (23.84, 27.76) | 25.95 (24.23, 28.17) | 0.078 |
| Risk factors |  |  |  |
| Cigarette smoking, n (%) | 712 (60.14) | 146 (63.48) | 0.381 |
| Diabetes, n (%) | 482 (40.71) | 115 (50.00) | 0.011 |
| Hypertension, n (%) | 761 (64.27) | 145 (63.04) | 0.779 |
| Dyslipidemia, n (%) | 1180 (99.66) | 230 (100.00) | 0.838 |
| Previous MI, n (%) | 150 (12.67) | 57 (24.78) | <0.001 |
| Previous stroke, n (%) | 137 (11.57) | 24 (10.43) | 0.702 |
| Previous PCI, n (%) | 276 (23.31) | 120 (52.17) | <0.001 |
| PAD, n (%) | 152 (12.84) | 42 (18.26) | 0.037 |
| Family history of CAD, n (%) | 219 (18.50) | 41 (17.83) | 0.883 |
| Clinical presentations |  |  |  |
| Multi-vessel CAD, n (%) | 943 (79.65) | 197 (85.65) | 0.044 |
| LVEF (%) | 64 (61, 66) | 63 (60, 66) | 0.044 |
| Laboratory measurements |  |  |  |
| TC (mmol/L) | 4.03±1.03 | 4.18±1.08 | 0.047 |
| LDL-C (mmol/L) | 2.40±0.84 | 2.51±0.90 | 0.054 |
| HDL-C (mmol/L) | 1.10±0.31 | 1.06±0.26 | 0.058 |
| Triglycerides (mmol/L) | 1.73±1.22 | 1.90±1.15 | 0.047 |
| FPG (mmol/L) | 6.18±2.04 | 6.75±2.52 | <0.001 |
| HbA1c (%) | 6.32±1.13 | 6.68±1.47 | <0.001 |
| Pcr, (μmol/L) | 81.42±15.83 | 82.40±25.61 | 0.444 |
| eGFR, (ml/min per 1.73m^2^) | 110.46 (97.07, 124.41) | 114.52 (96.86, 129.85) | 0.117 |
| NT-proBNP, (pg/ml) | 164.70±345.32 | 173.29±275.97 | 0.722 |
| hs-CRP, (mg/L) | 4.06±5.88 | 4.02±6.22 | 0.923 |
| Medications at discharge |  |  |  |
| DAPT, n (%) | 1181 (99.75) | 230 (100.00) | 1.000 |
| Statins, n (%) | 1159 (97.89) | 227 (98.70) | 0.586 |
| Dual-lipid lowering therapy, n (%) | 48 (4.05) | 14 (6.09) | 0.229 |
| ACEI/ARBs, n (%) | 578 (48.82) | 129 (56.09) | 0.052 |
| β-blockers | 975 (82.35) | 208 (90.43) | 0.003 |
| Insulin, n (%) | 117 (9.88) | 38 (16.52) | 0.005 |
| Oral hypoglycemic drugs, n (%) | 290 (24.49) | 77 (33.48) | 0.006 |
| Angiographic findings |  |  |  |
| Restenotic lesions, n (%) | 36 (3.04) | 37 (16.09) | <0.001 |
| Chronic total occlusions, n (%) | 149 (12.58) | 42 (18.26) | 0.028 |
| Lesions>20 mm long, n (%) | 825 (69.68) | 175 (76.09) | 0.061 |
| Number of stents | 2 (1, 2) | 2 (1, 3) | 0.071 |
| Length of stent, (mm) | 30.00 (21.00, 46.00) | 35.00 (23.00, 56.75) | 0.007 |
| TyG index | 8.82 (8.46, 9.21) | 9.00 (8.58, 9.43) | <0.001 |
| TyG tertiles |  |  | <0.001 |
| T_1_, n (%) | 408 (87.37) | 59 (12.63) |  |
| T_2_, n (%) | 399 (85.62) | 67 (14.38) |  |
| T_3_, n (%) | 377 (78.38) | 104 (21.62) |  |

ACEI, angiotensin-converting enzyme inhibitor; ARBs, angiotensin receptor blockers; BMI, body mass index; CAD, coronary artery disease; DAPT, dual antiplatelet therapy; eGFR, estimated glomerular filtration rate; FPG, fasting plasma glucose; HDL-C, high-density lipoprotein-cholesterol; HbA1c, glycated hemoglobin A1c; hs-CRP, hypersensitive C-reactive protein; LVEF, left ventricular ejection fraction; LDL-C, low-density lipoprotein-cholesterol; MI, myocardial infarction; NT-proBNP, N-terminal pro-B-type natriuretic peptide; PCI, percutaneous coronary intervention; PAD, peripheral arterial disease; Pcr, plasma creatine; TC, total cholesterol; TyG, triglyceride–glucose.

**Table S3**. Angiographic findings and discharge medications of the study patients according to tertiles of the TyG index

| Variable | T_1_ (n=467) | T_2_ (n=466) | T_3_ (n=481) | P-value |
| --- | --- | --- | --- | --- |
| Angiographic findings |  |  |  |  |
| One-vessel disease, n (%) | 102 (21.84) | 87 (18.67) | 85 (17.67) | 0.239 |
| Two-vessel disease, n (%) | 146 (31.26) | 147 (31.55) | 153 (31.81) | 0.984 |
| Three-vessel/LM disease, n (%) | 218 (46.68) | 234 (50.21) | 243 (50.52) | 0.425 |
| Target vessel territory |  |  |  |  |
| LM, n (%) | 17 (3.64) | 10 (2.15) | 17 (3.53) | 0.340 |
| LAD, n (%) | 268 (57.39) | 251 (53.86) | 278 (57.80) | 0.410 |
| LCX, n (%) | 117 (25.05) | 118 (25.32) | 117 (24.32) | 0.935 |
| RCA, n (%) | 173 (37.04) | 199 (42.70) | 171 (35.55) | 0.059 |
| Trifurcation/bifurcation lesions, n (%) | 255 (54.60) | 238 (51.07) | 280 (58.21) | 0.088 |
| Restenotic lesions, n (%) | 20 (4.28) | 26 (5.58) | 27 (5.61) | 0.576 |
| Chronic total occlusions, n (%) | 53 (11.35) | 74 (15.88) | 64 (13.31) | 0.127 |
| Calcification lesions, n (%) | 211 (45.18) | 215 (46.14) | 216 (44.91) | 0.924 |
| LAD proximal lesions, n (%) | 200 (42.83) | 218 (46.78) | 216 (44.91) | 0.478 |
| Lesions>20 mm long, n (%) | 321 (68.74) | 335 (71.89) | 344 (71.52) | 0.511 |
| TIMI grade 0/1, n (%) | 73 (15.63) | 96 (20.60) | 90 (18.71) | 0.141 |
| Number of stents | 2 (1, 2) | 2 (1, 2.75) | 2 (1, 2) | 0.076 |
| Length of stent, (mm) | 30.00 (20.00, 45.50) | 33.00 (21.00, 51.00) | 30.00 (22.00, 46.00) | 0.231 |
| Stent diameter, (mm) | 2.75 (2.50, 3.50) | 2.75 (2.50, 3.00) | 2.75 (2.50, 3.00) | 0.043 |
| Medications at discharge |  |  |  |  |
| DAPT, n (%) | 464 (99.36) | 464 (99.57) | 476 (98.96) | 0.523 |
| Statins, n (%) | 454 (97.22) | 458 (98.28) | 474 (98.54) | 0.301 |
| Dual-lipid lowering therapy, n (%) | 15 (3.21) | 19 (4.08) | 28 (5.82) | 0.135 |
| ACEI/ARBs, n (%) | 199 (42.61) | 244 (52.36) | 264 (54.89) | <0.001 |
| β-blockers | 372 (79.66) | 393 (84.33) | 418 (86.90) | 0.009 |
| Insulin, n (%) | 36 (7.71) | 32 (6.87) | 87 (18.09) | <0.001 |
| Oral hypoglycemic drugs, n (%) | 74 (15.85) | 100 (21.46) | 193 (40.12) | <0.001 |
| Metformin, n (%) | 25 (5.35) | 36 (7.73) | 77 (16.01) | <0.001 |
| Alpha-glucosidase inhibitors, n (%) | 51 (10.92) | 62 (13.30) | 119 (24.74) | <0.001 |
| Sulfonylurea, n (%) | 23 (4.93) | 31 (6.65) | 57 (11.85) | <0.001 |
| DPP-4 inhibitors, n (%) | 10 (2.14) | 19 (4.08) | 28 (5.82) | 0.016 |

ACEI, angiotensin-converting enzyme inhibitor; ARBs, angiotensin receptor blockers; DAPT, dual antiplatelet therapy; DPP-4, dipeptidyl peptidase-4; LM, left main coronary artery; LAD, left anterior descending coronary artery; TyG, triglyceride–glucose; TIMI, thrombolysis in myocardial infarction.

**Table S4.** Associations between the TyG index and repeat revascularization

|  | Model 1 | | | Model 2 | | | Model 3 | | |
| --- | --- | --- | --- | --- | --- | --- | --- | --- | --- |
|  | HR | 95%CI | P-value | HR | 95%CI | P-value | HR | 95%CI | P-value |
| TyG index | 1.276 | 1.109-1.468 | <0.001 | 1.192 | 1.032-1.377 | 0.017 | 1.181 | 1.022-1.366 | 0.024 |
| TyG tertiles |  |  |  |  |  |  |  |  |  |
| T_1_ | Reference |  |  | Reference |  |  | Reference |  |  |
| T_2_ | 1.353 | 1.081-1.693 | 0.008 | 1.241 | 0.987-1.560 | 0.065 | 1.223 | 0.973-1.538 | 0.085 |
| T_3_ | 1.414 | 1.134-1.764 | 0.002 | 1.292 | 1.030-1.622 | 0.027 | 1.274 | 1.015-1.600 | 0.037 |
| P for trend |  |  | 0.002 |  |  | 0.031 |  |  | 0.042 |

Model 1: unadjusted

Model 2: adjusted for age, sex, BMI, previous PCI, presence of PAD, presence of multivessel CAD, high-sensitivity CRP, eGFR, presence of lesion’s length ≥ 20 mm

Model 3: adjusted for age, sex, BMI, previous PCI, presence of PAD, presence of multivessel CAD, high-sensitivity CRP, eGFR, presence of lesion’s length ≥ 20 mm, stent length.

BMI, body mass index; CI, confidence interval; CAD, coronary artery disease; CRP, C-reactive protein; eGFR, estimated glomerular filtration rate; HR, hazard ratio; PCI, percutaneous coronary intervention; PAD, peripheral artery disease; TyG, triglyceride-glucose.

**Table S5.** Associations between the TyG index and in-stent restenosis

|  | Model 1 | | | Model 2 | | | Model 3 | | |
| --- | --- | --- | --- | --- | --- | --- | --- | --- | --- |
|  | HR | 95%CI | P-value | HR | 95%CI | P-value | HR | 95%CI | P-value |
| TyG index | 1.496 | 1.223-1.830 | <0.001 | 1.442 | 1.176-1.768 | <0.001 | 1.423 | 1.159,1.748 | <0.001 |
| TyG tertiles |  |  |  |  |  |  |  |  |  |
| T_1_ | Reference |  |  | Reference |  |  | Reference |  |  |
| T_2_ | 1.143 | 0.806-1.623 | 0.453 | 1.127 | 0.788-1.611 | 0.513 | 1.107 | 0.774-1.583 | 0.578 |
| T_3_ | 1.806 | 1.312-2.486 | <0.001 | 1.774 | 1.277-2.464 | <0.001 | 1.738 | 1.250-2.417 | 0.001 |
| P for trend |  |  | <0.001 |  |  | <0.001 |  |  | <0.001 |

Model 1: unadjusted

Model 2: adjusted for age, sex, BMI, previous PCI, presence of PAD, presence of multivessel CAD, high-sensitivity CRP, eGFR, presence of lesion’s length ≥ 20 mm

Model 3: adjusted for age, sex, BMI, previous PCI, presence of PAD, presence of multivessel CAD, high-sensitivity CRP, eGFR, presence of lesion’s length ≥ 20 mm, stent length.

BMI, body mass index; CI, confidence interval; CAD, coronary artery disease; CRP, C-reactive protein; eGFR, estimated glomerular filtration rate; HR, hazard ratio; PCI, percutaneous coronary intervention; PAD, peripheral artery disease; TyG, triglyceride-glucose.
